# Endophytic Bacteria as Modulators of Fragrance Biosynthesis in Plants: Mechanisms and Prospects

**DOI:** 10.3390/ijms27219950

**Published:** 2026-11-06

**Authors:** Priyanshi Srivastava, Sunil Kumar Verma, Juveriya Israr, Sankalp Misra, Manish Kumar, Diby Paul

**Affiliations:** 1 Faculty of Biosciences, Institute of Biosciences and Technology, Shri Ramswaroop Memorial University, Lucknow-Deva Road, Barabanki 225003, Uttar Pradesh, India; 2 Faculty of Biotechnology, Institute of Biosciences and Technology, Shri Ramswaroop Memorial University, Lucknow-Deva Road, Barabanki 225003, Uttar Pradesh, India; 3 Amity Institute of Biotechnology, Amity University, Noida 201313, Uttar Pradesh, India; 4 College of Life and Health Sciences, Truett McConnell University, Cleveland, GA 30528, USA

**Keywords:** endophytic bacteria, plant fragrance, volatile organic compounds, plant–microbe interactions, microbial elicitation, aromatic plants, metabolic engineering

## Abstract

Plant fragrance is the blend of volatile terpenoids, phenylpropanoids/benzenoids, fatty acid derivatives, and nitrogen- or sulfur-containing compounds released from flowers, leaves, and fruit. It drives pollination and defence and accounts for much of the economic value of aromatic, medicinal, and ornamental crops. However, it shifts with genotype, developmental stage, tissue type, and stress, and a large share of it still comes from wild-harvested plants. Endophytic bacteria are the microorganisms that colonize internal plant tissues without causing apparent disease symptoms, may modulate these specialized metabolic pathways. Only a limited number of studies have directly investigated these interactions. In contrast, a substantially larger body of research has demonstrated the roles of endophytic bacteria in promoting plant growth and enhancing plant tolerance to environmental stresses. However, direct evidence establishing links between bacterial endophytes, specific genes within volatile-biosynthetic pathways, and the volatile compounds actually released by plants remains limited. The current review summarizes the current evidence on the regulation of major plant volatile biosynthetic routes by endophytic bacteria, including the MVA and MEP pathways for terpenoids, the shikimate-derived phenylpropanoid/benzenoid pathway, as well as the lipoxygenase pathway leading to green-leaf volatile production. The suggested mechanisms are generally grouped into several inter-related categories, such as microbial elicitation and pattern recognition, phytohormonal reprogramming, nutrient and precursor acquisition, modulation of carbon flux through the MVA/MEP and phenylpropanoid pathways, redox regulation and stress priming, and bacterial volatile-mediated signalling. More speculative mechanisms are discussed separately, such as microbial biotransformation, direct microbial biosynthesis of plant volatiles, and horizontal gene transfer. Evidence from culinary and essential oil herbs, medicinal plants, ornamental flowers, and fruit crops is evaluated using an evidence hierarchy that distinguishes phenotypic associations from experimentally validated causal mechanisms. Establishing a robust gene-to-metabolite causal framework will require targeted approaches, including gnotobiotic inoculation, volatilomic profiling, and stable-isotope tracing.

## 1. Introduction

Fragrance originates when a plant’s volatile organic compounds (VOCs), small, lipophilic molecules that evaporate easily, pass through its tissues and escape into the air. These VOCs are emitted from multiple organs, including roots, stems, leaves, fruits, and seeds. In many flowering species, floral tissues exhibit specialized biosynthetic allocation and regulated volatile emission linked to pollinator attraction. Floral scent contributes to pollinator attraction and orientation toward floral rewards. Volatiles emitted from leaves and flowers also participate in direct and indirect defence against herbivores, including repellence and the recruitment of natural enemies [1,2]. Some of these compounds additionally contribute to pathogen defence, interplant signalling, and reproductive success.

Plant-derived fragrance compounds have substantial commercial importance. Perfumery, cosmetics, pharmaceuticals, food flavouring, aromatherapy, and essential oils depend on plant scent chemistry. In addition, the plants themselves are traded worldwide, with “natural” ingredients more in demand than ever. However, increasing demand is exerting considerable pressure on wild populations, particularly slow growing and narrowly distributed species, with many aromatic and medicinal plants traded globally now facing varying levels of conservation concern [2,3]. This is an increasing problem, however, as the demand for natural fragrances grows while their plant sources are finite and increasingly threatened by climate change and overharvesting. These pressures have sparked increasing interest in biological approaches to fragrance production as alternatives to traditional chemical synthesis and genetic engineering.

The richness of plant scents comes from different groups of compounds that have different biosynthetic pathways and precursor molecules. The largest and most varied group of these is terpenes based on the five-carbon isoprene. The group includes the two main groups of terpenes: monoterpenes and sesquiterpenes, in addition to higher terpenes [2]. Another group is phenylpropanoids and benzenoids that are derived from the amino acid phenylalanine [1,4]. This group is the second most chemically rich and its components include compounds like methyl benzoate and eugenol. The third group concerns fatty acids. The oxidative breakdown of membrane lipids gives rise to different compounds like aldehydes and alcohols [2]. Volatiles that contain nitrogen and sulfur may be few in number, but their influence on ecosystems and odors is significant. The metabolism of these four classes makes use of various substrates: terpenoids utilize acetyl-CoA and pyruvate/glyceraldehyde-3-phosphate, phenylpropanoids and benzenoids utilize phenylalanine, and oxylipins utilize the fatty acids of the membranes. Microbiota-associated changes in host primary metabolism may therefore influence precursor availability and volatile production across multiple compound classes.

Fragrance yield and composition depend heavily on plant genotype, developmental stage, tissue type, and growing conditions. Essential-oil composition also shifts from season to season and from one growing region to another, even within a single cultivar. Baseline essential-oil yields are often low, and some fragrance compounds break down chemically once they have formed, so a good share of what a plant biosynthesizes never makes it to commercial extraction. Environmental stresses like drought, heat, salinity, pathogen pressure, and nutrient deficiency can each push essential-oil composition in a different direction, potentially increasing or decreasing essential-oil yield and altering its chemical composition, and which way it goes depends on the species and the severity and duration of stress exposure. These limitations have important agronomic, commercial, and conservation implications. Many of the most valuable aromatic and medicinal plants are still harvested wild, at least in part, and overharvesting has already pushed several traditionally wild-collected species toward threatened status. Therefore, the case for better agronomy isn’t just about increasing essential-oil yield per plant, or getting it more consistently. Improved cultivation practices may also reduce reliance on wild harvesting to meet commercial demand.

Endophytic bacteria live inside plant roots, stems, leaves, flowers, and seeds for some or all of their life cycle, usually without causing disease. The bacterial cell stays in constant molecular contact with living host tissue, which sets endophytes apart from epiphytes on the plant surface and from the broader rhizosphere community [5]. That distinction is why so much has been written on how endophytes affect plant growth, nutrition, stress tolerance, hormone balance, and primary and specialized metabolism [6,7]. Bacterial endophytes specifically have also been tied to shifts in terpenoid, phenolic, flavonoid, and essential-oil accumulation in aromatic and medicinal species. What is harder to find is evidence that actually connects a given endophyte to a specific fragrance-pathway gene, enzyme, or emitted compound, rather than to the usual, often confounded, changes in plant biomass, nutrition, or stress physiology. That’s the gap this review is aimed at: a wide, mostly correlative literature on endophytes and secondary metabolism, set against a much narrower and more mechanistic one on growth promotion and induced resistance [8,9]. However, direct evidence linking a specific endophyte to fragrance-pathway genes, enzyme activities, or emitted volatiles remains restricted to individual pathway components, rather than generalized shifts in plant biomass, nutrition, or stress physiology. This review addresses this gap by bridging a broad, largely correlative literature on endophytes and secondary metabolism with a more focused, mechanistic understanding of growth promotion and induced resistance.

This review also traces the biochemical pathways behind plant fragrance and the mechanisms by which endophytic bacteria are reported to shift them. It assesses evidence across culinary herbs, essential-oil crops, medicinal plants, ornamentals, and fruit crops, though the extent and mechanistic resolution vary by crop group. Although this review primarily focuses on endophytic bacteria that colonize internal plant tissues, it also considers selected studies on epiphytic and phyllosphere-associated bacteria as comparative references where they provide relevant insights into microbial modulation of plant volatile profiles. These microbial groups occupy distinct ecological niches and should not be treated as interchangeable. However, comparisons across these habitats can help identify shared or contrasting mechanisms affecting volatile biosynthesis, accumulation, and emission. A central challenge is to move beyond correlation toward causal understanding through integrated analytical, genomic, and synthetic-biology approaches. The review highlights the remaining knowledge gaps and defines the research requirements for developing a mechanistic framework for bacteria-mediated fragrance biotechnology.

## 2. Plant Fragrance Biosynthesis: A Biochemical Foundation

### 2.1. Terpenoid Biosynthesis

**Figure 1:**
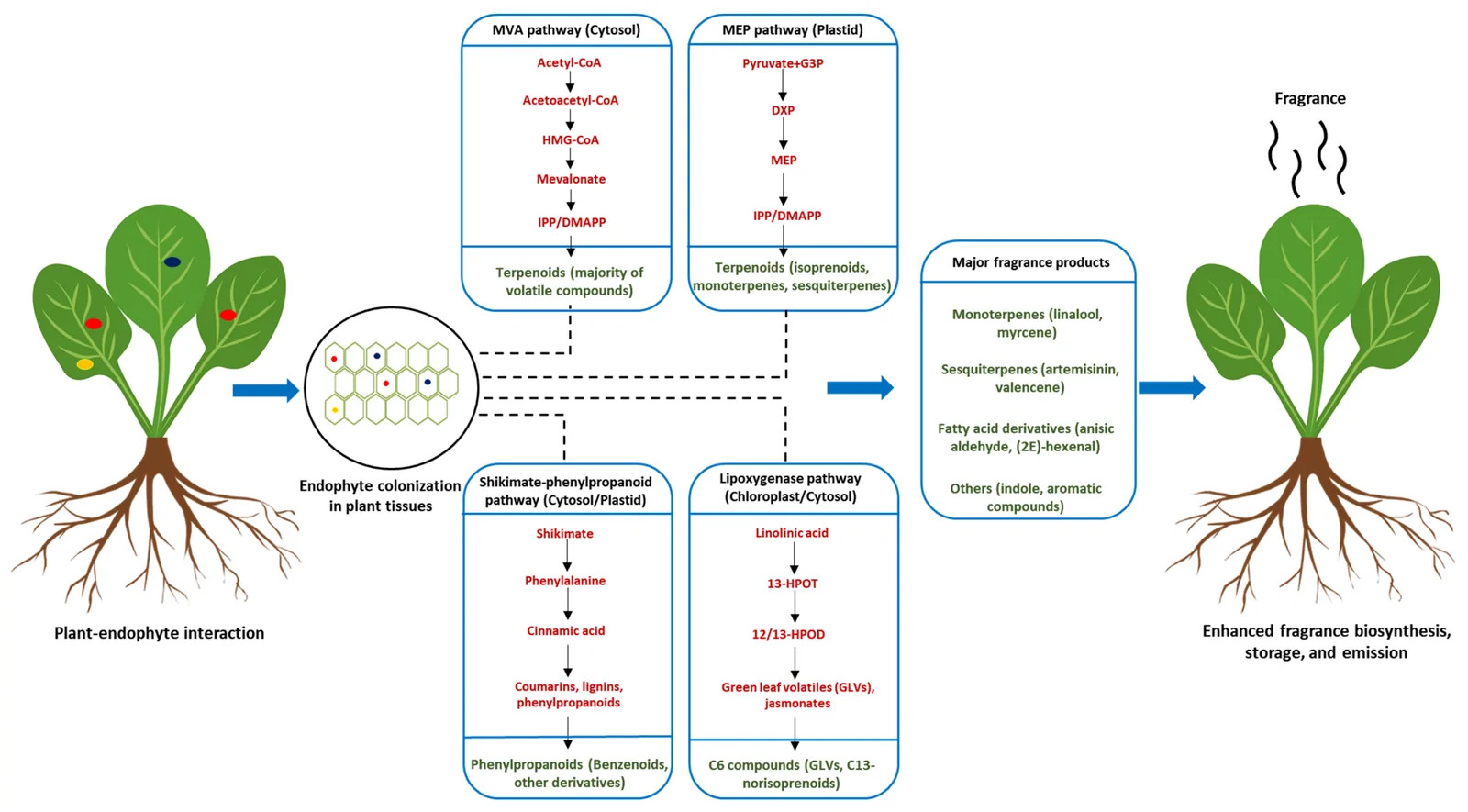
A schematic illustration indicating the significance of endophytes in the fragrance production. The endophytic invasion leads to change in the many metabolic processes that are responsible for synthesizing volatile compounds and their accumulation. The mevalonate (MVA) pathway in the cytosol and the methylerythritol phosphate (MEP) pathway in plastids generate isoprenoid precursors, including isopentenyl diphosphate (IPP) and dimethylallyl diphosphate (DMAPP), which contribute to the biosynthesis of monoterpenes and sesquiterpenes. The shikimate–phenylpropanoid pathway produces phenylpropanoids, benzenoids, coumarins, and related derivatives, whereas the lipoxygenase pathway converts linolenic acid into green leaf volatiles, jasmonates, and C_6_/C_13_ norisoprenoid compounds. These pathways collectively generate major fragrance constituents, including linalool, myrcene, artemisinin, valencene, anisic aldehyde, (2E)-hexenal, indole, and other aromatic compounds. Endophyte–plant interactions may therefore enhance fragrance biosynthesis, tissue-level storage, and volatile emission, resulting in the observed fragrance phenotype. Abbreviations: DXP, 1-deoxy-D-xylulose 5-phosphate; G3P, glyceraldehyde 3-phosphate; HMG-CoA, 3-hydroxy-3-methylglutaryl-CoA; IPP, isopentenyl diphosphate; DMAPP, dimethylallyl diphosphate; MEP, 2-C-methyl-D-erythritol 4-phosphate; 13-HPOT, 13-hydroperoxyoctadecatrienoic acid; 12/13-HPOD, 12/13-hydroperoxyoctadecadienoic acid; GLVs, green leaf volatiles.

Every terpenoid starts from the same two five-carbon units: isopentenyl diphosphate (IPP) and its isomer, dimethylallyl diphosphate (DMAPP) (Figure 1). Plants have two entirely separate ways of making them, occurring in two different parts of the cell. In the cytosol, it is the mevalonate (MVA) pathway, with HMGR (3-hydroxy-3-methylglutaryl-CoA reductase) running the rate-limiting step. In plastids, a second pathway does the same job by a different route: methylerythritol phosphate (MEP), kicked off by DXS and DXR (1-deoxy-D-xylulose-5-phosphate synthase and reductoisomerase) (Figure 1). These compartmentalized pathways provide distinct routes for the synthesis of the shared isoprenoid precursors IPP and DMAPP. Prenyltransferases link IPP and DMAPP head-to-tail in systematic condensation reactions to build longer chains—GPP (C10), FPP (C15), and GGPP (C20)—that act as the direct building blocks for monoterpenes, sesquiterpenes, and diterpenes, respectively. Terpene synthases (TPSs) subsequently convert these prenyl diphosphate substrates into diverse terpene structures (Figure 1). This is an unusually large and functionally diverse family of enzymes, and among them, they cyclize and rearrange these chains into whatever ring structure gives a given species, or even a single tissue, its signature terpene. The resulting products may undergo further modification by carrying out their own transformations, oxidizing or decreasing the product, and in some cases shutting it down completely. The most significant aspect of this last stage is the fact that it is what establishes the volatility, stability, and storage characteristics of products [2,3].

### 2.2. Phenylpropanoid and Benzenoid Biosynthesis

Phenylalanine, which is created through the shikimate pathway, is the precursor to both benzenoids and phenylpropanoids. The phenylalanine ammonia-lyase (PAL) enzyme removes the amino group, producing trans-cinnamic acid. After this stage of production, a number of enzymes, namely, C4H and 4CL, will convert trans-cinnamic acid into not only benzaldehyde and benzoic acid, but also the essential substances for producing scents, namely, methyl benzoate, methyl salicylate, eugenol, isoeugenol, and vanillin [1]. The process of producing scent is not an isolated one, as the same pool of phenylalanine is a source for making both lignin and flavonoids. Furthermore, many enzymes acting here, namely, methyl and acyltransferases, act in the process of producing phenolic compounds (Figure 1). Thus, it is necessary to increase flux to produce either lignin or flavonoids, as otherwise there will be insufficient phenylalanine to be used to produce fragrances. This might be the reason why the production of fragrances and production of protective compounds is interconnected [4].

### 2.3. Fatty-Acid-Derived and Green-Leaf Volatiles

Fatty-acid-derived volatile biosynthesis represents another major route of plant aroma formation and can be rapidly activated following tissue damage. Lipoxygenases are enzymes that convert polyunsaturated fatty acids in cell membranes into other fatty acid compounds. Hydroperoxide lyase cuts the broken down fatty acids into six-carbon aldehydes, such as hexanal and (Z)-3-hexenal, which are responsible for the cut-grass odor. Alcohol dehydrogenase is able to convert these six-carbon compounds into alcohols like the (Z)-3-hexen-1-ol compound, while the alcohol acyltransferase processes them into esters. This pathway can be rapidly activated following mechanical wounding or herbivory, resulting in the release of green-leaf volatiles (Figure 1) [2].

### 2.4. Compartmentation, Transport, Storage, and Emission

The production of aroma compounds is arranged on different levels in their development. Inside the cell the MEP pathway operates in the plastid, while the MVA and phenylpropanoids pathways take place in the cytoplasm. Since the pathways are distributed among various compartments, the transporters are responsible for transfer of intermediate compounds between them. In some plants, both terpenoid pathways communicate as well. These processes affect whether the substances make it to the actual scent. The separation occurs at the tissue level as well. Numerous aromatic plants have specialized secretory tissues which synthesize, store and emit their flavoring components. These tissues include glandulae found on leaves, osmophores in flowers, and cavities producing oils throughout the bark, leaves and pericarp. However, if a plant has already synthesized a certain compound, it does not necessarily mean that the substance is available to leave the plant’s interior. Very often, aromatic oils are found in a glycoside form, that needs to be oxidized before the smell can be released. This hydrolysis process can be treated as a release checkpoint. Apart from the above process, volatile emission is also subject to temporal regulation. In many species, floral scent emission is developmentally regulated and follows circadian patterns at certain stages of their life cycle, especially when they are blooming, and also take into account their circadian rhythm, in order to release the scent when the pollinators come out [3].

### 2.5. Transcriptional and Hormonal Regulation

Several transcription-factor families coordinate expression of fragrance-pathway genes: MYB, basic helix-loop-helix (bHLH), APETALA2/ethylene-response factor (AP2/ERF), WRKY, NAC, and basic leucine zipper (bZIP) proteins. These act alone and in combinatorial complexes to activate terpene synthase, PAL, and other modifying-enzyme promoters in a tissue- and stage-specific manner. These transcriptional programmes are themselves under hormonal control [2,10]. Jasmonic acid, salicylic acid, ethylene, abscisic acid, auxin, and cytokinin all feed into fragrance-pathway regulation, tying volatile output to the same signalling networks that govern defence, developmental timing, and stress responses. Upstream regulation also involves rapid signalling events: calcium fluxes, reactive oxygen species (ROS), mitogen-activated protein kinase (MAPK) cascades, and shifts in redox state. These signalling events provide a potential mechanistic basis for endophytic bacterial modulation of fragrance biosynthesis [11].

## 3. Endophytic Bacteria as Modulators of Aroma and Fragrance in Plants

### 3.1. Diversity and Colonization Niches

Culturable and metagenomic surveys of aromatic and medicinal plants recover bacterial endophytes from a recurring set of genera, including *Bacillus*, *Pseudomonas*, *Paenibacillus*, *Enterobacter*, *Burkholderia* and related genera, *Streptomyces* and other actinobacteria, and *Methylobacterium* and other facultative methylotrophs [6]. These taxa have been isolated repeatedly from oregano, basil, and strawberry, among many other hosts, and they colonize roots, stems, leaves, flowers, seeds, and species that possess glandular trichomes and other specialized secretory tissues [8,9,12]. Colonization of secretory structures is of particular interest for fragrance research because it places bacterial cells in direct physical proximity to the very tissues responsible for terpenoid and phenylpropanoid biosynthesis and storage.

### 3.2. Factors Shaping Endophyte–Host Associations

Which bacterial taxa establish themselves, and how densely, depends on plant genotype and chemotype, tissue age and developmental stage, prevailing environmental conditions, the composition of the surrounding soil and rhizosphere microbial community, and agricultural management practices, as well as on the host’s nutritional and physiological status. Endophyte transmission can occur vertically, through seed, or horizontally, by recruitment from the rhizosphere and phyllosphere, and the balance between these routes helps determine how consistently a given endophyte–host association reappears across generations and growing sites [6].

### 3.3. Endophytes as Producers, Modifiers, or Elicitors of Fragrance Compounds

Three potential functional roles can be distinguished, and they are not mutually exclusive. An endophyte may be a direct producer of flavor compounds, as scientists have already recorded free-living, plant-associated bacteria creating complex terpenes. On the other hand, it may act as a biotransformer, processing precursors created by the plant or freeing aroma compounds contained in pre-formed glycosides [13]. That seems to be the role played by a phyllosphere methylotroph, which generates furanoid flavor components in strawberry tissues. Alternatively, the bacterium could simply influence the plant’s metabolism via elicitation, hormone signalling, or nutritional supplies [12]. Insights from genome mining of oregano endophytes suggest that these bacteria have some MEP pathway and auxin-biosynthesis genes, but not all of the genes necessary for a complete terpene synthase system [14]. Distinguishing bacterial production from host-mediated changes in aroma metabolism is important for mechanistic interpretation and biotechnological application. When a host starts to produce more aromas, the reason could be that either the plant’s own metabolic pathway was triggered or the microbes directly affected the plant’s volatile content, or the plant had been producing such compounds all along but simply got better at releasing them. Few studies have experimentally distinguished these contributions with relevant methodological approaches, as discussed in further sections.

## 4. Mechanisms of Endophytic Bacterial Modulation

Endophytic bacteria are unlikely to influence fragrance biosynthesis through a single, universal mechanism. Instead, the literature points to a set of interacting mechanistic modules that operate with different weight in different plant–bacterium combinations. This section reviews each module in turn.

### 4.1. Microbial Elicitation and Molecular Recognition

**Figure 2:**
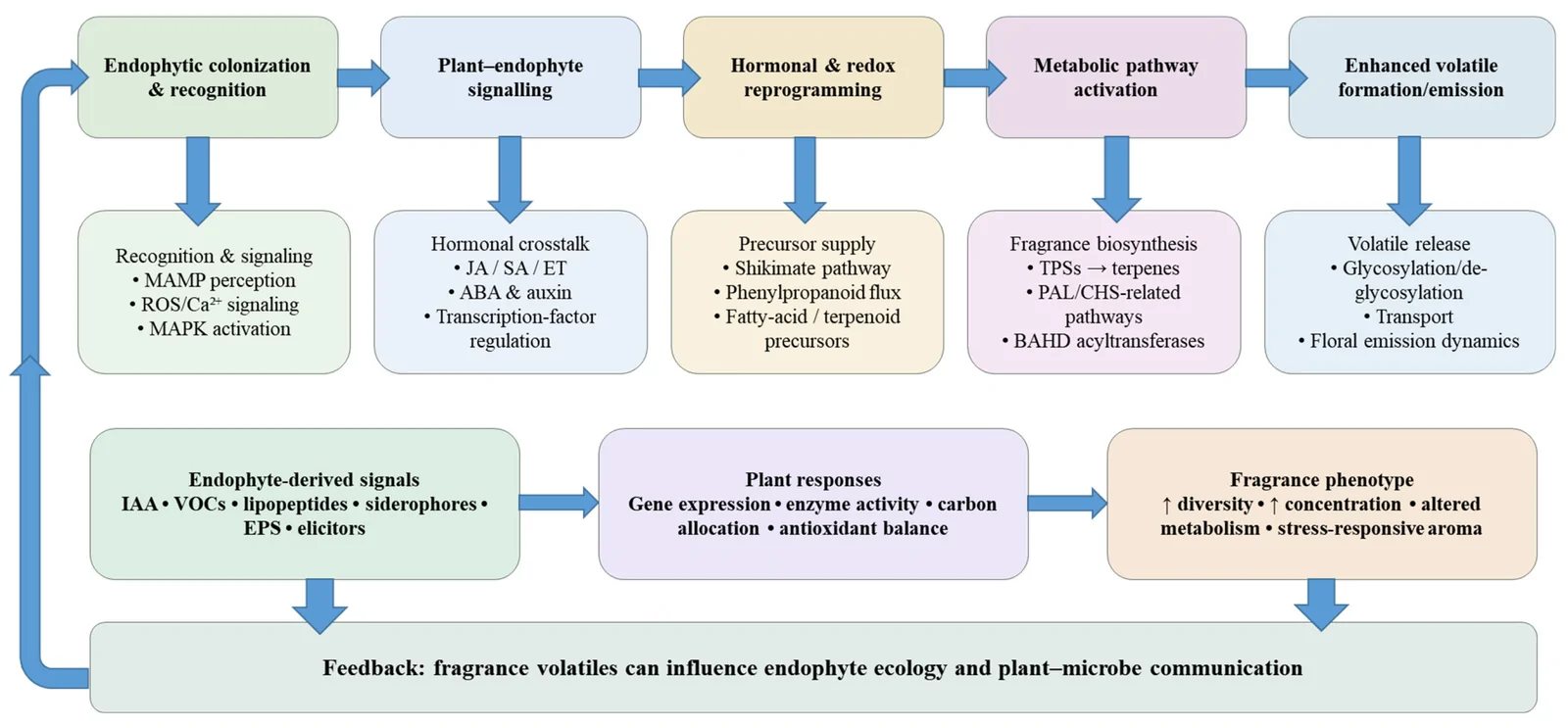
Flowchart illustrating sequential molecular events linking endophyte colonization to floral/foliar volatile output. Endophytic colonization and recognition initiate plant–endophyte signalling through MAMP perception, ROS/Ca^2+^ signalling, and MAPK activation. Endophyte-derived signals, including indole-3-acetic acid (IAA), volatile organic compounds (VOCs), lipopeptides, siderophores, exopolysaccharides (EPS), and other elicitors, promote hormonal crosstalk involving jasmonic acid (JA), salicylic acid (SA), ethylene (ET), abscisic acid (ABA), and auxin, together with transcription-factor regulation. Subsequent hormonal and redox reprogramming enhances precursor supply through the shikimate, phenylpropanoid, fatty-acid, and terpenoid pathways, thereby activating fragrance biosynthesis via terpene synthases (TPSs), phenylalanine ammonia-lyase (PAL)/chalcone synthase (CHS)-associated pathways, and BAHD acyltransferases. These processes influence glycosylation/de-glycosylation, volatile transport, and floral emission dynamics, ultimately generating a fragrance phenotype characterized by altered volatile diversity, concentration, metabolism, and stress-responsive aroma profiles. The figure also depicts plant responses involving changes in gene expression, enzyme activity, carbon allocation, and antioxidant balance, as well as feedback from fragrance volatiles to endophyte ecology and plant–microbe communication.

Bacterial cells produce a range of conserved molecular signals including lipopolysaccharides, flagellin and flagellin-derived peptides, peptidoglycan fragments, exopolysaccharides, siderophores, cyclic lipopeptides, bacterial VOCs, and secreted enzymes or effector-like molecules—that plant cells detect through surface-localized pattern-recognition receptors [11]. Recognizing these microbe-associated molecular patterns (MAMPs) sets off a stereotyped signalling sequence: within seconds to minutes, receptor activation drives a calcium influx and a burst of reactive oxygen species, which together activate mitogen-activated protein kinase (MAPK) cascades [10] (Figure 2). These cascades phosphorylate and activate downstream transcription factors. Several of the transcription-factor families this cascade recruits—MYB, bHLH, and WRKY proteins among them—also regulate terpene synthase and phenylpropanoid-pathway promoters (Section 2.5). These overlapping regulatory networks provide a potential mechanistic connection between microbial recognition and fragrance-pathway regulation [11].

### 4.2. Hormonal Reprogramming

Endophytic bacteria can promote changes in the hormonal balance of their hosts, some of whose hormones also regulate fragrance biosynthesis. An example is peppermint, where inoculation with these bacteria resulted in a several-fold rise of levels of both jasmonic acid and salicylic acid while at the same time enhancing essential-oil content and the density of glandular trichomes. The effects of applying jasmonate and salicylate to fruit-bearing plants corroborate some of the findings. The part played by hormones in such regulation is not uniform across all bacterial strains [15]. Different strains are capable of activating different branches of the pathway; for example, in tomato, one particular growth-promoting rhizobacterium activated only the salicylic acid pathway while another one worked through the jasmonate/ethylene branch, resulting in different physiological and defense responses of the same plant [16]. The actions of auxin and cytokinin as hormones are also responsible for regulating the trichome structure, oil glands, and osmophores. Abscisic-acid signalling takes care of the response to stress: it links endophyte colonization of plants with the chemistry of the volatiles produced in response to drought and salinity, as evidenced by damask rose plants inoculated with endophytes under salt stress conditions [17].

### 4.3. Nutrient Acquisition and Precursor Availability

A second mechanism functions indirectly through the nutrient state of the plant; many endophytes render their host a more nutrient-rich organism through nitrogen fixation, solubilization of phosphate, iron uptake using siderophores, and better assimilation of sulfur, as a result of which the pool of primary metabolites (acetyl-CoA, pyruvate, glycerol for glycolysis, and aromatic amino acids) is increased. In basil (*Ocimum basilicum*), strains of *Microbacterium foliorum* and *Paenibacillus peoriae* known for their phosphate solubilization and beneficial traits for plant growth raised the concentration of essential oil by up to 100% and increased the amounts of chlorophyll, carotenoids, and anthocyanin. Because of the increase in pigments, one might be able to conclude that the primary metabolism was intense rather than think that it is a cause of special features of the fragrance process [8]. Since the mechanism is located upstream of the pathway-specific gene regulation, it might be difficult to distinguish it from the real activation of the genes involved in the fragrance production during an experiment.

### 4.4. Regulation of MVA and MEP Pathways

Where gene-expression data exist, endophyte inoculation changes transcription of core isoprenoid-pathway genes. In *Withania somnifera*, endophyte inoculation altered expression of squalene synthase, squalene epoxidase, HMG-CoA reductase, and farnesyl pyrophosphate synthase, and these shifts tracked tissue-specific changes in withanolide (a sterol-derived terpenoid) content in leaves and roots [7]. Separately, genome mining of oregano endophytes found that most of these bacteria carry a complete, bacterial-type MEP/DOXP pathway and can make their own IPP and DMAPP (Figure 2). The interaction may therefore extend beyond the regulation of host gene expression [14]. A viable conceptual framework is that colonization alters the carbon flow across growth, primary metabolism, defense, and fragrances, with the balance being dictated by the strain, host genotype, and the environment.

### 4.5. Activation of Phenylpropanoid and Benzenoid Pathways

Endophytes have also been associated with increased production of volatiles derived from benzenoids and phenylpropanoids, although the relevant enzymes are known far less than terpenoid genes, which include phenylalanine ammonia-lyase, cinnamate-4-hydroxylase, 4-coumarate-CoA ligase, and various methyltransferases and acyltransferases for the production of benzoates, eugenol, and other esters (Figure 2). In damask rose, colonization increased phenylethyl alcohol and oxygenated monoterpenoid volatiles, such as citral and citronellol; however, the effect varied by cultivar [17]. Terpenoid and phenylpropanoid volatiles tend to shift together rather than shift between two pathways sequentially.

### 4.6. Stress Priming and Redox Regulation

Different endophytic bacteria can prepare the host plant for drought, high temperatures, salinity, and pollution by heavy metals, oxidative activity, disease-causing microorganisms, and herbivores. For instance, in salt-stressed damask rose, inoculation reduced oxidative damage and maintained the activity of antioxidant metabolism; moreover, this protective effect was associated with the change in floral volatile content in comparison to stressed, uninoculated plants. Since ROS, the activity of antioxidant enzymes, and redox balance contribute to the process of fragrance synthesis, it is highly likely that stress preparation enables a plant to change scented compounds used for its chemical communication, even if the exact mechanism of this influence remains unknown [17].

### 4.7. Bacterial VOC-Mediated Effects

Bacteria associated with plants, including endophytes, emit volatile substances like sulfur compounds, alcohols, terpenes, and ketones that can influence the physiology of the pathogen without any direct contact. A well-characterized example is 2,3-butanediol and acetoin produced by *Bacillus subtilis* GB03 managed to stimulate the growth of Arabidopsis even when applied as airborne substances; when mutants that cannot produce these VOCs are used, however, the stimulatory effect is lost [18]. Furthermore, the concept of induced systemic resistance, ethylene signalling alteration, and iron uptake enhancement are also included in this process, but the extent of these effects varies considerably and strongly depends on the experimental conditions [19]. As relevant to the topic of this review, epiphytic and floral bacteria can contribute it, initiate it, suppress it, or simply degrade the isolated floral volatiles produced by the control flowers—and thus change the fragrance of the flower for which the pollinators come. Collectively, these findings indicate that plant-associated bacteria can influence host gene expression, induce growth, affect systemic resistance, have an influence on the terpene synthase process, and serve as a means of communication among different kingdoms [20].

### 4.8. Direct Microbial Biosynthesis and Biotransformation

Another less explored option is that endophytic bacteria contribute directly to aroma production by producing terpenes and aromatics by themselves, processing plant precursors into easily volatile substances, deglycosylating precursors, or transforming alcohols, aldehydes, acids, and esters. For instance, *Serratia plymuthica*, which is a rhizobacterium, associated with plants and which has a distinct terpenoid cyclase gene for the production of the rare terpenoid sodorifen. This proves that free-living plant-colonizing bacteria may possess the machinery required for terpenoid synthesis [13]. An example of a biotransformation-type instance is represented by the methylotrophic bacterium *Methylobacterium extorquens*, which is responsible for an increased production of compounds that produce fragrance for strawberries. However, these findings require cautious interpretation: in most field and greenhouse studies, bacterial abundance correlating with fragrance-compound levels is not the same as proving the bacterium made the compound. This section keeps the rare cases with real biochemical evidence separate from the larger body of evidence based primarily on correlative observations [12].

### 4.9. Horizontal Gene Transfer and Shared Biosynthetic Potential

A further, currently speculative mechanism is that biosynthetic genes move between plant-associated microbes, or even between microbes and their hosts, changing what an endophyte population can make over time. Comparative genomics of plant-associated bacteria does turn up terpene-family biosynthetic gene clusters—plus NRPS and RiPP clusters—often enough, and distributed unevenly enough across taxa, to suggest a real reservoir of specialized-metabolite potential in these communities. Direct evidence demonstrating any specific cluster moved to or from a plant host, as opposed to horizontal transfer among bacterial lineages, is less reported. However, pending strain-resolved genomic analyses and functional validation, claims in this area should stay cautious.

### 4.10. Epigenetic and Post-Transcriptional Regulation

The potential contribution of epigenetic and post-transcriptional regulation to endophyte-mediated changes in fragrance biosynthesis remains insufficiently characterized. Candidate mechanisms include DNA methylation, histone modification, chromatin remodelling, and small-RNA-mediated regulation of genes involved in terpene and phenylpropanoid biosynthesis. Although these regulatory processes have been implicated in plant stress responses, their specific roles in endophyte-mediated modulation of fragrance pathways require targeted investigation.

## 5. Evidence from Important Plant Systems

Rather than working through individual studies plant by plant, this section groups the evidence by crop type and ends with a table applying a common evidence hierarchy across systems.

### 5.1. Culinary and Essential-Oil Plants

The herbs from the Lamiaceae family, including basil, mint, rosemary, thyme, oregano, and lavender, have been the subject of many studies focused on the influence of endophytes on the characteristics of essential oils, which can be due, among other reasons, to the presence of glandular trichomes in these plants, allowing for easy measurement of their content and composition. In the case of oregano, endophytes originating from the above-mentioned parts of the plant were able to produce volatile compounds that have the same type as the essential oil of the plant, namely monoterpenes (like α-pinene, 3-carene, p-cymene, γ-terpinene), as well as dimethyl disulfide and dimethyl trisulfide. In addition, some of the isolates were also shown to have antibacterial properties against pathogenic bacteria [9]. In-depth examination of the collection of the above-mentioned endophytes showed that in each of the strains, genes responsible for the MEP/DOXP pathway for isoprenoid precursors as well as for the biosynthesis of indolylacetic acid were found, which suggests that the main mechanism is not the specific terpenoid pathway but rather precursor supply and growth stimulation [14]. In basil, two isolates namely *Microbacterium foliorum* and *Paenibacillus peoriae*, raised essential-oil content by roughly 50–100%, along with gains in chlorophyll, carotenoids, anthocyanins, and antioxidant activity. In peppermint, plant growth-promoting bacteria raised endogenous jasmonic and salicylic acid, increased glandular trichome density, and boosted essential-oil yield; bacterial inoculation and exogenous jasmonate application together produced more than either alone [8,15]. Outcomes measured across these systems typically include oil yield, monoterpene/sesquiterpene profiles, biomass, trichome density, and—less often—pathway gene expression.

### 5.2. Medicinal and Aromatic Plants

Several medicinal plants provide useful evidence that endophytic bacteria can influence terpenoid-related secondary metabolism, although the metabolites investigated are not necessarily fragrance compounds. In *Withania somnifera*, bacterial endophytes were reported to alter the tissue-specific accumulation and biosynthetic localization of withanolides, including withaferin A and withanolide A, alongside changes in the expression of genes associated with sterol and triterpenoid biosynthesis, such as HMGR, FPPS, squalene synthase, and squalene epoxidase [7]. Withanolides are C28 steroidal lactones with pharmaceutical relevance rather than volatile aroma constituents. Similarly, in Artemisia annua, the endophytic actinobacterium *Pseudonocardia* sp. strain YIM 63111 was associated with increased expression of CYP71AV1 and cytochrome P450 oxidoreductase and enhanced accumulation of artemisinin [21]. Artemisinin is a non-volatile sesquiterpene lactone used in antimalarial therapy, not a fragrance volatile. These studies therefore provide evidence that endophytes can regulate terpenoid-related biosynthetic pathways and specialized metabolism, but they should not be interpreted as direct demonstrations of altered fragrance biosynthesis or volatile emission. Because these pathways share upstream precursors with some volatile terpenoids, the findings offer relevant mechanistic context for fragrance research; direct relevance to plant scent, however, requires volatile profiling and/or emission measurements in fragrance-producing systems. These distinctions also emphasize that changes in plant growth, pharmaceutical metabolites, and fragrance-related volatiles may not occur in parallel [22].

### 5.3. Ornamental and Fragrant Flowers

The availability of direct evidence from scent-based ornaments may be limited, but it is steadily developing. It has been shown that inoculating damask rose (*Rosa damascena*) with *Bacillus pumilus* or *Vibrio campbellii* altered the composition of floral volatiles in a cultivar-dependent manner, specifically influencing the concentrations of phenylethyl alcohol and oxygenated monoterpenes such as citronellol, citral, and neral. Inoculated plants were less affected by oxidative stress and maintained a better functioning of antioxidant metabolism [17]. In the study involving *Brassica rapa* species, the flowers were shown to differ in their volatiles’ profile depending on whether they were colonized by epiphytic bacteria or remained sterile; in other words, the presence of bacteria led to the emergence of novel compounds, or to changes in the production of some other compounds. For conceptual clarity, the *Brassica rapa* example is considered here as a comparative reference rather than direct evidence of endophytic bacterial activity, because the study examined floral-associated epiphytic bacteria. Although this work demonstrates that bacteria inhabiting the flower surface can influence floral volatile profiles, its findings cannot be directly attributed to internal endophytes. Nevertheless, it provides useful comparative evidence that plant-associated bacteria can influence volatile composition within the floral environment. That is evidence that floral bacteria causally reshape the volatile blend reaching pollinators, not just a correlation. Outcomes of interest here include volatile emission rate and composition, flower-opening timing, specific pollinator-attracting compounds, the benzenoid-to-terpenoid balance, and circadian emission rhythms [20].

### 5.4. Food and Fruit Crops

In fruit crops, microbial associations have been linked to flavor-relevant volatile chemistry, particularly in strawberry. The phyllosphere-associated methylotroph *Methylobacterium extorquens* DSM 21961 was associated with increased biosynthesis of 2,5-dimethyl-4-hydroxy-2H-furanone and its methoxy derivative, both important contributors to strawberry aroma [12]. This example is included as comparative evidence of microbial modulation of aroma-related metabolism in a plant-associated habitat. However, the findings should not be interpreted as direct evidence of endophyte-mediated fragrance modulation.

### 5.5. Comparative Synthesis of Available Evidence

The available evidence using a four-level hierarchy, ranging from fragrance-related phenotypic changes (Level I) to progressively stronger molecular, metabolomic, and functional evidence (Levels II–IV) summarized in Table 1. The evidence must, however, be interpreted in relation to the metabolite actually measured. Studies involving Mentha × piperita provide evidence relevant to essential-oil production and monoterpene accumulation, whereas studies involving *Withania somnifera* and *Artemisia annua* demonstrate endophyte-associated regulation of non-volatile specialized metabolites, namely withanolides and artemisinin, respectively. The latter studies support the broader proposition that endophytic bacteria can influence terpenoid-related metabolism because some upstream precursor pathways overlap with those involved in volatile terpenoid biosynthesis, but they do not constitute direct evidence of altered fragrance volatile composition or emission. Accordingly, the evidence levels assigned to these medicinal-plant studies should be understood as reflecting the molecular and metabolite evidence available for the specific compounds investigated, rather than as equivalent evidence for fragrance modulation. Direct validation of fragrance-related effects requires appropriate volatile profiling and, where relevant, measurements of emission rates. This distinction is important when comparing mechanistic findings across medicinal, aromatic, ornamental, and food plants. An important distinction must be maintained between essential-oil accumulation and volatile emission when interpreting the effects of bacterial inoculation. Essential-oil yield determined by hydrodistillation or other extraction methods reflects the amount of recoverable oil in the sampled plant material, whereas headspace-based analyses characterize volatile compounds released into the surrounding atmosphere under defined sampling conditions. An increase in extracted oil yield therefore does not necessarily indicate a proportional increase in atmospheric fragrance emission, because release is also influenced by compound-specific volatility, tissue storage, transport, environmental conditions, and emission dynamics. Accordingly, studies reporting increased essential-oil yield are interpreted here as evidence of enhanced oil accumulation, while claims concerning fragrance emission are reserved for studies that directly measure headspace volatile profiles or emission rates.

## 6. Methodological Approaches for Endophyte Selection

### 6.1. Characterization of Bacterial Endophytes

Culture-dependent isolation of endophytes requires proper surface-sterilization procedures followed by plating the sterilization effluent to confirm no contamination. While 16S rRNA gene sequencing remains the standard method for genus- and species-level taxonomic identification, whole-genome sequencing is increasingly used to resolve strain identity and screen for functional traits. Genome-based surveys of oregano-associated Bacillus endophytes provide a clear illustration of this dual approach [14,21]. Culture-independent techniques, including metagenomics and metatranscriptomics, are able to detect organisms that do not grow under culture conditions. In addition, one of the benefits of these techniques is that they can reveal gene expression in plants. Fluorescent tagging can provide direct spatial evidence of endophytic colonization. For example, Li et al. (2012) [29] used an enhanced green fluorescent protein (EGFP)-expressing *Pseudonocardia* sp. strain YIM 63111 and confocal laser-scanning microscopy to visualize intercellular colonization of internal root tissues in *Artemisia annua*. These observations complemented re-isolation of the inoculated strain [8].

### 6.2. Inoculation and Experimental Designs

Experimental systems can be as diverse as strictly axenic and gnotobiotic cultures or hydroponic and growth-chamber arrangements to greenhouse trials and, occasionally, field experiments. A tradeoff occurs between control and realism as one moves along this continuum from axenic systems which allow one to claim the phenotype in question belongs to a particular strain of microorganisms to field experiments which take into account the full abundance of the local microbiome, soil chemistry and climate and make causal attribution exceptionally difficult. The experimental procedure in terms of what the researcher is actually testing also varies between studies—whether single strains are compared with untreated controls, whether one tests synthetic communities made from known components, or whether the researcher measures activity in living or nonliving samples [30].

### 6.3. Fragrance and Volatile Analysis

The selection of analytical method is critical to the interpretation of fragrance-related responses. Headspace solid-phase microextraction coupled with gas chromatography–mass spectrometry (HS-SPME–GC–MS) provides an approach for sampling and characterizing plant volatiles with limited disturbance to the sampled tissue. Dynamic headspace sampling and 2D GC-MS can further improve upon this by giving better sensitivity, accuracy and resolution in analysis of complex mixtures. GC–olfactometry provides complementary information on the sensory relevance of detected compounds as it indicates corresponding peaks to odour-active compounds [31]. Meanwhile, PTR-ToF-MS even allows monitoring emissions in real time. Hydrodistillation is widely used to extract and quantify essential oils; however, it measures extractable oil content rather than ambient volatile emissions. Stable-isotope labelling and chiral analysis are underutilized approaches with potential value for mechanistic analysis [32].

### 6.4. Molecular and Biochemical Analyses

qPCR and RNA-seq remain the main tools for tracking fragrance-pathway gene expression—used, for example, to follow withanolide-pathway genes in *Withania somnifera* and CYP71AV1/CPR expression in *Artemisia annua*. Proteomics and direct enzyme assays check whether those transcriptional changes actually translate into altered enzyme activity, because transcriptional changes do not necessarily correspond to changes in enzyme abundance or activity [29]. Hormone profiling and measurements of reactive oxygen species and antioxidant enzymes are needed to test the hormonal-reprogramming and stress-priming mechanisms discussed in previous sections. The jasmonate/salicylate measurements taken alongside essential-oil quantification in peppermint represent an illustrative exampleReporter-gene assays, promoter analysis, gene-editing or silencing validation of candidate transcription factors, and transcriptomic profiling of the bacterium during colonization provide additional complementary approaches for moving from correlation to mechanism [15].

### 6.5. Metabolomics and Flux Analysis

Untargeted metabolomics and targeted volatile profiling work best paired with analysis of non-volatile precursors and glycosidic storage forms. A change in headspace volatile concentration could mean altered biosynthesis, altered hydrolysis of stored glycosides, or altered emission, and those three have very different mechanistic implications, so failure to distinguish these processes may lead to incorrect mechanistic attribution [33]. Stable-isotope tracing and formal metabolic flux analysis are still rare in this literature. They are also the clearest way to quantify how much carbon from a fragrance compound actually passes through a bacterially influenced step. Correlation networks linking bacterial abundance, host gene expression, and fragrance chemistry are useful for generating mechanistic hypotheses that can subsequently be evaluated through isotope tracing and functional experiments [34].

### 6.6. Establishing Correlation

A central methodological consideration is that correlation between bacterial presence and a host phenotype does not establish evidence of causation. Synthetic-community and gnotobiotic approaches from the broader field offer a template—reconstitute a defined bacterial community in a gnotobiotic host, add or remove individual strains or genes, and read out a quantitative phenotype [35]. Applied specifically to fragrance biology, establishing causality requires re-isolation and authentication of the endophyte following experimentation, confirmation of in planta colonization using appropriate colonization controls, and the use of heat-killed cells and purified elicitor controls to distinguish effects of live bacterial colonization from those mediated by diffusible signals. These further validations would best use bacterial mutants deficient in candidate traits, such as the production of volatile organic compounds, siderophore biosynthesis, or elicitation activities, followed by genetic complementation to determine whether the phenotype is restored. These experiments should be complemented with validations in microbe-free, defined gnotobiotic systems, gene function studies in the host by silencing, genome editing, or construction of mutant plant lines, to follow carefully designed time-course and dose-response experiments to differentiate between early elicitation responses from slowly cumulative nutritional effects [36].

## 7. Factors Affecting the Outcome of Bacterial Inoculation

The outcome of the inoculation is determined primarily by a combination of host genotype and chemotype, the developmental stage of the plant at the time of inoculation, the particular plant tissues colonized by the bacterium, the number of glandular trichomes or other secretory structures present, the plant’s endogenous microbiome, and its nutrient condition. Host genotype and chemotype are important determinants of inoculation outcomes. Two cultivars of damask rose, given the same inoculum, showed different floral-volatile levels, as host genotype is an equally important factor [37]. On the bacterial side, the characteristics of the strain, its efficacy in colonization and growth, and its ability to produce biofilms and how this affects the process of endophyte development. The way the strain functions with regard to different biological compounds is important for establishing a connection with different pathways discussed in Section 4. Being compatible with a particular strain is not sufficient to ensure that the strain will remain stable after being introduced into a host [17].

Temperature, light and length of day, water availability, soil characteristics, nutrients, salinity, pressure from pathogens and CO_2_ in the atmosphere influence the physiology of the plants and the activity of the bacteria—and many factors that influence oil composition independently of any biological effect. This is the main reason why responses observed under greenhouse conditions may not be consistently reproduced under field conditions. Most of the discrepancies are thought to have been caused by differences in the density of inoculum, method of application, timing and frequency of inoculation, substrate, harvest time, extraction method, and lastly the platform used [38]. As essential oil yield and composition are strongly influenced by a number of these factors, regardless of the biological effect, cross-study comparisons including the Table 1 should be interpreted as informative rather than as precise comparisons.

## 8. Applications and Biotechnological Prospects

For biotechnological applications, essential-oil yield and atmospheric fragrance emission should be treated as distinct production outcomes. Endophyte-mediated increases in extractable essential oil may improve the quantity of material available for processing, but they do not directly demonstrate enhanced fragrance release from living plants or harvested products. Conversely, changes in headspace volatile composition or emission rates may occur without a corresponding increase in total extracted oil yield. Therefore, claims of improved essential-oil productivity should be supported by extraction-based measurements, whereas claims of enhanced fragrance emission should be supported by appropriate headspace analysis and, where feasible, quantitative emission measurements. Where both outcomes are relevant, complementary analytical approaches can provide a more complete assessment of the effects of microbial inoculation.

### 8.1. Sustainable Production of Essential Oils and Natural Fragrances

The most apparent application is agronomic: plant varieties that consistently interact to increase essential-oil yield per unit biomass, as seen with basil and mint, can help to increase crop value without increasing land use, while also reducing the use of fertilizers and pesticides where these same strains lead to higher biomass or provide protection from pests. Because several of the mechanisms in Section 4.2 and Section 4.6 work through stress-tolerance pathways, endophyte-based approaches might help stabilize oil production under the drought, heat, and salinity affecting aromatic-crop production in many regions. That could open marginal land to high-value aromatic crops that may otherwise be economically unsuitable for cultivation [39].

### 8.2. Improvement of Fragrance Quality

**Figure 3:**
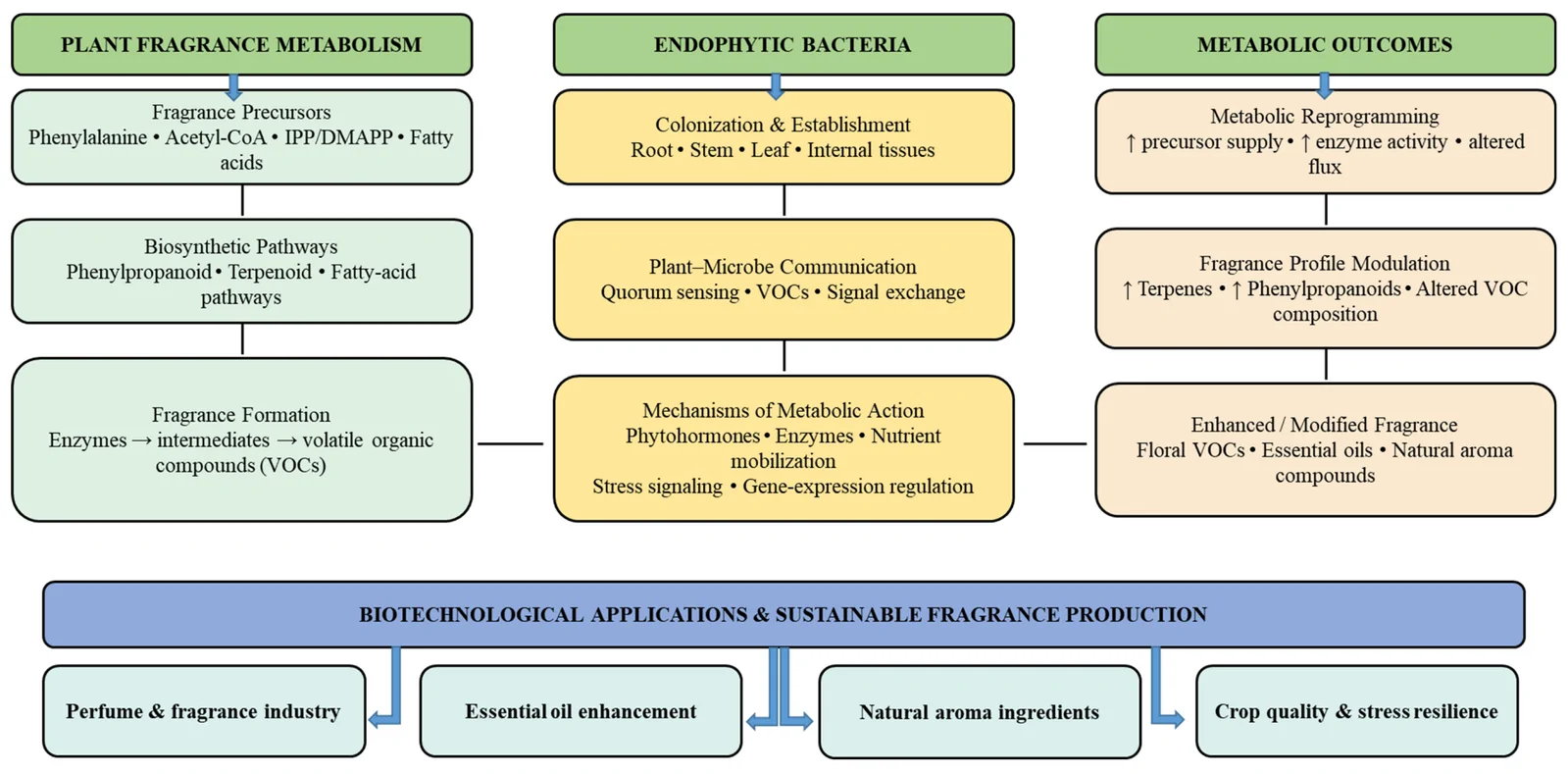
Integrative framework of endophytic bacteria-mediated modulation of plant fragrance metabolism and its biotechnological implications.

Beyond yield, selected endophytes could improve quality directly: more of the desirable aroma compounds, less of the unwanted or allergenic ones, subtle chemotype shifts, or enhancement of specific enantiomers or notes that carry outsized sensory value—the strain- and cultivar-dependent shifts in monoterpenoid and benzenoid composition seen in inoculated rose point in this direction. A less obvious benefit would be better batch-to-batch consistency, which is an important limitation of wild-harvested and open-field aromatic crops (Figure 3).

### 8.3. Microbial Consortia and Synthetic Communities

Different endophytic strains tend to specialize—one in nutrient provision, another in hormone modulation, a third in elicitor production—which is a reasonable argument for building consortia with complementary functions instead relying exclusively on a single multifunctional strain. Community-level approaches, borrowing synthetic-community methodology from the wider plant-microbiome field, could improve colonization robustness and functional stability under the field conditions under which single-strain inoculants may exhibit inconsistent establishment or performance.

### 8.4. Plant Tissue Culture

Plant cell, tissue, and organ cultures provide controlled platforms for investigating and producing specialized metabolites. Hairy-root cultures have been investigated as production systems for bioactive compounds, including cultivation in bioreactors. For example, Kim et al. (2001) [39] compared nutrient mist and bubble-column reactors for *Artemisia annua* hairy roots and demonstrated reactor-dependent differences in artemisinin accumulation. These findings support the evaluation of hairy-root systems for controlled metabolite production, although the effects of endophytic bacteria or their purified elicitors on fragrance-related pathways in such systems require direct experimental validation.

### 8.5. Metabolic Engineering and Synthetic Biology

Some bacteria clearly demonstrate their own biosynthetic capability. Sodorifen production in *Serratia plymuthica* serves as a primary example of this dedicated terpene synthase activity. Future investigations should evaluate the feasibility of transferring this biosynthetic module to a more tractable heterologous host. Alternatively, research could focus on engineering the native endophyte to achieve a predictable and controllable yield. Pairing conventional breeding with deliberate microbiome engineering [40], and developing plant-associated microbial chassis for sustained in planta production, are longer-term extensions of the mechanistic work in Section 4, but potentially high-value ones [41].

### 8.6. Climate-Resilient Aromatic Agriculture

Abiotic stress already hurts both yield and chemical consistency in many traditionally grown aromatic species. Endophytes that maintain fragrance quality under drought, heat, salinity, or nutrient limitation—for example, bacterially inoculated roses kept their floral volatile and antioxidant profile under salt stress—could help stabilize production as growing conditions become less predictable [42] (Figure 3). Endophyte-assisted establishment on degraded or marginal soils could also ease pressure to expand cultivation, or wild collection, into new or sensitive areas, tying fragrance biotechnology to broader low-input and regenerative goals [43].

## 9. Limitations and Knowledge Gaps

Several limitations temper enthusiasm for the mechanisms and applications reviewed above. Few studies trace bacterial modulation of volatile biosynthesis down to the gene-to-metabolite level. Most investigations report macroscopic shifts in essential-oil yield or headspace composition without verifying pathway gene expression or enzymatic activity. Consequently, such studies align with Level I or II of the evidence hierarchy outlined in Section 5.5, rather than the higher-resolution Level III or IV. Reported interpretations do not always distinguish among the biological processes represented by the measured endpoints. Several studies determine total essential-oil content through the hydrodistillation technique rather than the real headspace emission in normal conditions, which means that what is stated as an increase in “fragrance” may be the result of more storage than actual emission. The problem is worsened by the fact that increased biomass after inoculation makes it appear that a bigger plant is producing more volatiles, although there is no demonstrated change in pathway-specific biosynthetic activity. Causal interpretations sometimes exceed the mechanistic resolution of the available evidence. A striking correlation between bacterial abundance and metabolite accumulation does not establish that one caused the other, and culture-dependent isolation is known to substantially underestimate endophytic diversity, meaning the taxa profiled in most studies are only a fraction of what is actually present. Strain effects are highly host- and environment-specific, and lab or greenhouse findings are rarely carried through to field-scale validation. Several additional issues remain insufficiently investigated. Long-term persistence and ecological safety of deliberately introduced strains remain underexplored. Sampling and reporting for plant fragrance still have no agreed standard across the literature. Single-strain inoculation studies tend to ignore whatever the native, non-inoculated community is doing and stereochemistry or sensory relevance rarely is assessed alongside chemical composition. As Section 4 and Section 6 note repeatedly, whether a metabolite is plant-produced or bacterially produced is often left unresolved.

## 10. Future Research Directions

The clearest way forward is to move beyond integrating indirect evidence from separate experimental systems on different plant-bacterium pairs—which is largely what the field has done so far—and instead run endophyte metagenomics, plant transcriptomics, proteomics, volatilomics, hormone profiling, epigenomics, and stable-isotope metabolomics within a coordinated experimental framework applied to the same plant–bacterium system. Fragrance biosynthesis, storage, and emission are compartmentalized down to the cell (Section 2.4), and whole-tissue extracts taken at one time point simply can’t resolve that. What’s needed is tissue-specific, ideally single-cell or spatially resolved, transcriptomics of endophyte colonization and host gene expression—trichome-associated bacteria profiled directly, not inferred—paired with tracking that actually follows flower development stage, circadian volatile shifts, and emission as it happens rather than after the fact.

In comparison to conventional methods, several recent approaches could substantially strengthen causal evidence. These include using bacterial mutants deficient in hormone, volatile organic compound (VOC), siderophore, or elicitor production; plant mutants with disrupted jasmonate, salicylate, MVA, MEP, or phenylpropanoid pathways; isotope tracing to determine whether bacterial carbon is incorporated into host fragrance compounds; in vitro reconstitution using purified enzymes; and synthetic microbial communities with predefined functional compositions, as outlined in Section 6.6. Once enough of these mechanistic datasets exist, predictive models linking specific bacterial traits to specific fragrance outcomes become plausible—machine learning could help narrow down which plant-bacterium combinations are worth testing before experimental validation. That points toward strain libraries organized by what a microbe actually does to fragrance rather than by taxonomy, and eventually toward models that fold in host genotype, environment, and community composition together. But greenhouse and gnotobiotic results are insufficient, on their own, to establish commercial feasibility. Getting there means multi-location field trials—checking whether effects hold up across seasons, whether they fit into how growers already farm, and whether there are unintended ecological effects—plus an rigorous evaluation of economic feasibility, formulation stability, and persistence of the desired effects and durable enough to be worth commercializing.

## 11. Conclusions

Endophytic bacteria represent an insufficiently characterized biological means of modulating plant fragrance. Across the plant–bacterium systems reviewed, changes in fragrance-related metabolism have been associated with microbial elicitation, hormonal regulation, nutrient and precursor availability, stress and redox responses, and bacterial volatile signalling. Direct bacterial biosynthesis and biotransformation provide additional potential contributions, although their importance within colonized plant tissues requires further validation. Despite increasing evidence from medicinal plants and essential-oil crops, few studies establish a continuous causal relationship between bacterial colonization, specific host or microbial pathways, and the production or emission of individual fragrance compounds. Future research should therefore combine gnotobiotic plant systems, bacterial genetics, stable-isotope tracing, transcriptomics, and high-resolution volatilomics to distinguish changes in biosynthesis from changes in storage, release, and plant biomass. Such mechanistic resolution will be essential for evaluating the reproducibility and biotechnological potential of endophyte-mediated fragrance modulation.

## Figures and Tables

**Table 1:** Comparative evidences for endophyte-associated modulation of plant volatile fragrance compounds and related specialized metabolites across representative plant systems.

Host Plant	Endophytic Bacterium	Plant Tissue	Main Compounds Measured	Proposed Mechanism	Analytical Method	Evidence Level	References
*Origanum vulgare* ssp. *vulgare* (oregano)	Culturable bacterial endophytes (multiple genera)	Aerial parts	Monoterpenes (α-pinene, 3-carene, p-cymene, γ-terpinene), dimethyl di-/trisulfide	VOC overlap between endophyte headspace and host essential oil; endophyte genomes carry MEP-pathway and IAA-biosynthesis genes	GC–MS; genome mining (KEGG, antiSMASH)	Level II	[9,23]
*Ocimum basilicum* (basil)	*Microbacterium foliorum* Emf1; *Paenibacillus peoriae* ER11	Whole plant/leaf	Essential oil (up to 101% yield increase)	Nutrient acquisition (P-solubilization, N-related traits) and general biostimulation of secondary metabolism	Hydrodistillation; phytochemical assays	Level I	[24]
*Mentha* × *piperita* (peppermint)	Consortium of plant growth-promoting bacteria (PGPB)	Leaf/glandular trichome	Total essential oil; monoterpenes (menthol, menthone)	Increased endogenous jasmonate and salicylate concentrations and glandular trichome density associated with enhanced essential-oil production	GC; phytohormone quantification	Level III	[15,25]
*Withania somnifera* (ashwagandha)	Root- and leaf-associated bacterial endophytes	Roots and leaves	Withanolides (e.g., withaferin A and withanolide A); non-volatile C28 steroidal lactones with pharmaceutical relevance, not fragrance volatiles	Altered expression of sterol/terpenoid-related pathway genes, including HMGR, FPPS, squalene synthase, and squalene epoxidase; relevant to broader terpenoid metabolism but not direct evidence of volatile fragrance modulation	HPLC; qRT-PCR of pathway genes	Level III (non-volatile specialized metabolism)	[7]
*Artemisia annua* (sweet wormwood)	*Pseudonocardia* sp. strain YIM 63111	Seedling tissue (confocal-confirmed colonization)	Artemisinin; a non-volatile sesquiterpene lactone with pharmaceutical relevance, not a fragrance volatile	Increased expression of CYP71AV1 and cytochrome P450 oxidoreductase, with increased artemisinin accumulation; evidence of specialized-metabolite pathway regulation rather than direct fragrance modulation	qRT-PCR; metabolite quantification	Level III (non-volatile specialized metabolism)	[26]
*Rosa damascena* (damask rose)	*Bacillus pumilus*; *Vibrio campbellii*	Floral tissue	Phenylethyl alcohol, citronellol, citral, neral (floral BVOCs)	Hormonal–redox modulation; maintenance of antioxidant metabolism under salinity	GC–MS; physiological/antioxidant assays	Level II	[27]
*Brassica rapa* (field mustard, model system)	Epiphytic bacterial assemblages (comparative evidence)	Flowers	Multiple floral VOCs (additions, reductions, and apparent catabolism of components)	Bacterial addition, induction, reduction, or catabolism of individual floral volatiles	Comparative GC–MS of sterile vs. inoculated flowers	Level I	[20]
*Fragaria* × *ananassa* (strawberry)	*Methylobacterium extorquens* DSM 21961 (phyllosphere-associated bacterium; comparative evidence)	Fruit/leaf surface	Furanoid flavour compounds (DMHF, DMMF)	In vitro biosynthesis/enhancement of furanone flavour precursors by methylotrophic metabolism	Real-time PCR strain monitoring; flavour-compound quantification	Level II	[28]

## Data Availability

Data sharing not applicable to this article as no datasets were generated or analyzed during the current study.
